# Norcembranoidal Diterpenes from a Formosan Soft Coral *Sinularia* sp.

**DOI:** 10.3390/molecules171214058

**Published:** 2012-11-27

**Authors:** Wei-Hsuan Yen, Li-Chung Hu, Jui-Hsin Su, Mei-Chin Lu, Wen-Hung Twan, Show-Ying Yang, Yung-Chi Kuo, Ching-Feng Weng, Chia-Hung Lee, Yueh-Hsiung Kuo, Ping-Jyun Sung

**Affiliations:** 1 Graduate Institute of Marine Biotechnology and Department of Life Science and Institute of Biotechnology, National Dong Hwa University, Pingtung 944, Taiwan; E-Mails: xyz78714@hotmail.com (W.-H.Y.); stoja582@gmail.com (L.-C.H.); x2219@nmmba.gov.tw (J.-H.S.); jinx6609@nmmba.gov.tw (M.-C.L.); cfweng@mail.ndhu.edu.tw (C.-F.W.); chlee016@mail.ndhu.edu.tw (C.-H.L.); 2 National Museum of Marine Biology and Aquarium, Pingtung 944, Taiwan; 3 Division of Marine Biotechnology, Department of Marine Biotechnology and Resources, Asia-Pacific Ocean Research Center, National Sun Yat-sen University, Kaohsiung 833, Taiwan; 4 Department of Life Science, National Taitung University, Taitung 950, Taiwan; E-Mail: twan@nttu.edu.tw; 5 Industrial Technology Research Institute, Clean Energy and Eco-Technology Center, Tainan 734, Taiwan; E-Mails: yangsy@itri.org.tw (S.-Y.Y.); itriA10063@itri.org.tw (Y.-C.K.); 6 Tsuzuki Institute for Traditional Medicine, China Medical University, Taichung 404, Taiwan; 7 Graduate Institute of Natural Products, Kaohsiung Medical University, Kaohsiung 807, Taiwan

**Keywords:** *Sinularia*, norcembranoidal diterpene, cytotoxicity

## Abstract

Two norcembranoidal diterpenes, 5-episinuleptolide acetate (**1**) and scabrolide D (**2**), were isolated from a Formosan octocoral identified as *Sinularia* sp. The structures of norcembranoids **1** and **2** were established by spectroscopic methods and by comparison of the spectral data with those of known analogues and **1** was proven to be a new natural product. Norcembranoid **1** was found to exhibit cytotoxicity toward a panel of tumor cells.

## 1. Introduction

**Figure 1:**
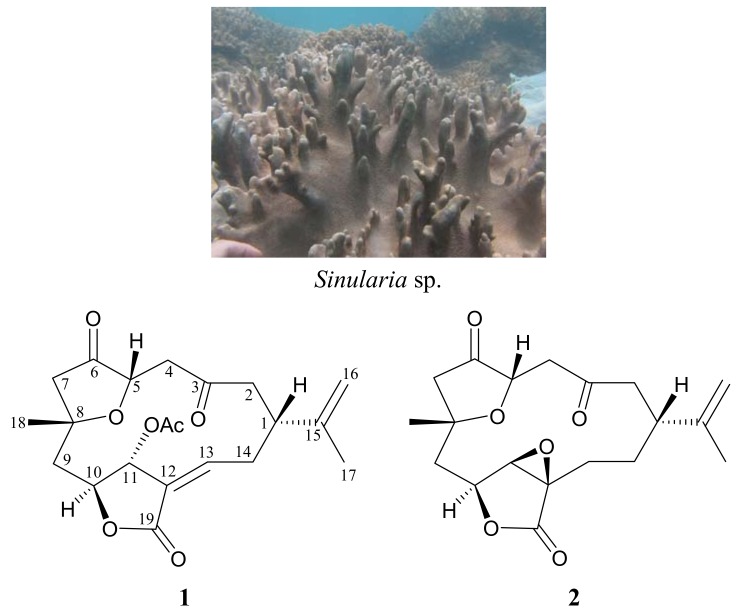
The soft coral *Sinularia* sp. and the structures of 5-episinuleptolide acetate (**1**) and scabrolide D (**2**).

The search for bioactive natural products from marine organisms has been remarkably successful [1,2] and octocorals belonging to the genus *Sinularia* have proven to be rich sources of bioactive terpenoid analogues [3]. Among these terpenoid metabolites, the C_19_-norcembranoid diterpene derivatives played an important role [4]. In continuation of our search for new natural substances from the marine invertebrates collected off the waters of Taiwan at the intersection of the Kuroshio current and the South China Sea surface current, we have further isolated two norcembranoidal diterpenes, 5-episinuleptolide acetate (**1**) and scabrolide D (**2**), from an octocoral identified as *Sinularia* sp. (Figure 1). In this paper, we describe the isolation, structure determination and cytotoxicity of norcembranoids **1** and **2**.

## 2. Results and Discussion

**Figure 2:**
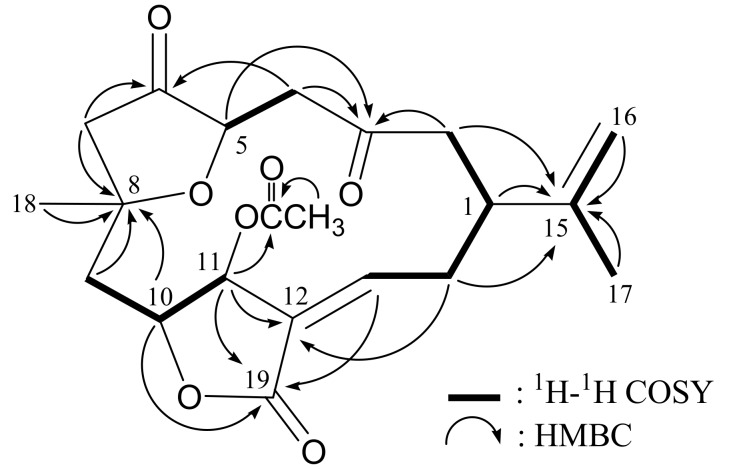
The ^1^H–^1^H COSY and key HMBC (protons→quaternary carbons) correlations for **1**.

The molecular formula for norcembranoidal diterpene **1** was determined to be C_21_H_26_O_7_ (nine units of unsaturation) using HRESIMS (C_21_H_26_O_7_+Na, *m/z* 413.1574, calculated 413.1576). The IR spectrum of **1** showed strong bands at 1756, 1738 and 1719 cm^–1^, consistent with the presence of ester and ketone carbonyl groups. From the ^1^H- and ^13^C-NMR spectra (Table 1), **1** was found to possess an acetoxy group (*δ*_H_ 2.08, 3H, s; *δ*_C_ 171.2, C; 20.9, CH_3_), an ester group (*δ*_C_ 167.7, C-19) and two ketone carbonyls (*δ*_C_ 205.3, C-3; 214.5, C-6). Two additional unsaturated functionalities were indicated by ^13^C resonances at *δ*_C_ 127.6 (C-12), 147.2 (CH-13), 147.0 (C-15) and 110.4 (CH2-16), suggesting the presence of a trisubstituted olefin and an exocyclic carbon-carbon double bond. From the ^1^H–^1^H COSY spectrum of **1** (Table 1 and Figure 2), it was possible to differentiate among the separate spin systems of H-1/H_2_-2, H_2_-4/H-5, H_2_-9/H-10/H-11, H-13/H_2_-14/H-1 and H_2_-16/H_3_-17 (by allylic coupling). These data, together with the key HMBC correlations between protons and quaternary carbons of **1** (Table 1 and Figure 2), such as H_2_-2, H_2_-4, H-5/C-3; H_2_-4, H_2_-7/C-6; H_2_-7, H_2_-9, H-10, H_3_-18/C-8; H-11, H_2_-14/C-12; H-1, H_2_-2, H_2_-14, H_2_-16, H_3_-17/C-15; and H-10, H-11, H-13/C-19, permitted the elucidation of the carbon skeleton. The acetoxy group positioned at C-11 was confirmed from the HMBC correlations of H-11 (*δ*_H_ 5.47) and protons of an acetate methyl (*δ*_H_ 2.08) to the ester carbonyl carbon at *δ*_C_ 171.2 (C). Thus, **1** was revealed as a norcembranoidal diterpene possessing a γ-lactone ring, on the basis of the above analysis. 

**Figure 3:**
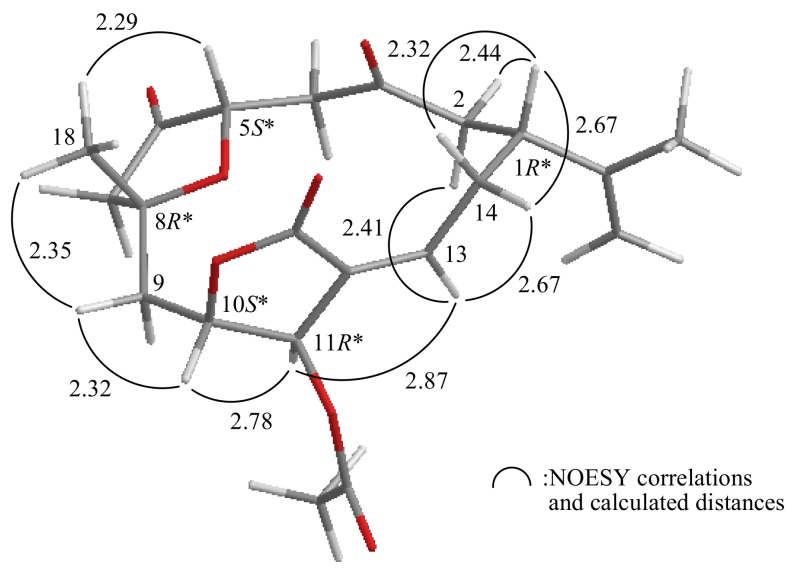
The computer-generated model of **1** using MM2 force field calculations and the calculated distances (Å) between selected protons with key NOESY correlations.

The relative configuration of **1** was elucidated mainly from a NOESY spectrum as being compatible with that of **1** offered by computer modeling (Figure 3), in which the close contacts of atoms in space calculated were consistent with the NOESY correlations [5]. Most naturally occurring cembrane-type natural products from soft coral belonging to the order Alcyonacea have the H-1 in the β-orientation [6]. In the NOESY experiment for **1**, H-1 correlated with H_2_-14 and one proton of C-2 methylene (*δ*_H_ 2.52, H-2β), supporting this reasoning by molecular modeling analysis. H-13 showed correlations with H-2α (*δ*_H_ 2.65), H-11 and one proton of C-14 methylene (*δ*_H_ 2.20, H-14α), but not with H-1, indicating the *E*-configuration of the C-12/13 double bond. H-10 showed a correlation with H-11, as well as the lack of coupling was detected between these two protons, indicating the dihedral angle between H-10 and H-11 is approximately 90° and the configurations of chiral carbons C-10 and C-11 were assigned as S*- and R*-forms, respectively. One proton of C-9 methylene (*δ*_H_ 2.47) correlated with H-10 and H_3_-18, but not with H-11, and H_3_-18 showed a strong correlation with H-5, indicating that Me-18 and H-5 were β-oriented in **1**. From the above evidences, the relative configuration of the chiarl carbons of **1** were assumed to be 1R*, 5S*, 8R*, 10S* and 11R*. 

By comparison of the spectral data with those of a known semisynthetic diterpene, compound **1** was elucidated unambiguously to be 5-episinuleptolide acetate [7]. To the best of our knowledge, norcembranoidal diterpene **1** was isolated for the first time from a natural source.

Norcembranoidial diterpene **2** was identified as scabrolide D, which had been isolated from a Formosan octocoral *Sinularia scabra*. Its spectral data were in full agreement with those previously reported [8]. 

Cytotoxicity of the norcembranoidal diterpenes **1** and **2** toward K562 (human erythromyeloblastoid leukemia), MOLT-4 (human acute lymphoblastic leukemia), HTC-116 (human acute promyelocytic leukemia), DLD-1 (human colorectal adenocarcinoma), T-47D (human breast ductal carcinoma) and MDA-MB-231 (human breast adenocarcinoma) cells was studied, and the results are shown in Table 2. These data showed that compound **1** (5-episinuleptolide acetate) exhibited modest cytotoxicity towards all the cells. 

## 3. Experimental

### 3.1. General Procedures

Optical rotation values were measured with a Jasco-P1010 digital polarimeter. Infrared spectra were obtained on a Varian Diglab FTS 1000 FT-IR spectrophotometer. NMR spectra were recorded on a Varian Mercury Plus 400 FT-NMR at 400 MHz for ^1^H and 100 MHz for ^13^C in CDCl_3_ at 25 °C. Proton chemical shifts were referenced to the residual CHCl_3_ signal (*δ*_H_ 7.26 ppm). ^13^C-NMR spectra were referenced to the center peak of CDCl_3_ at *δ*_C_ 77.1 ppm. ESIMS and HRESIMS data were recorded on Bruker APEX II mass spectrometer. Column chromatography was performed on silica gel (230–400 mesh, Merck, Darmstadt, Germany). TLC was carried out on precoated Kieselgel 60 F_254_ (0.25 mm, Merck) and spots were visualized by spraying with 10% H_2_SO_4_ solution followed by heating. HPLC was performed using a system comprised of a Hitachi L-7100 pump, a Hitachi L-7455 photodiode array detector and a Rheodyne 7725 injection port. A normal phase column (Hibar 250 × 10 mm, Merck, silica gel 60, 5 μm) was used for HPLC.

### 3.2. Animal Material

Specimens of the soft coral *Sinularia* sp. were collected by hand using scuba equipment off the coast of Sansiantai, Taitung County, Taiwan in October 13, 2011, and stored in a freezer (−20 °C) until extraction. This organism was identified by comparison with previous descriptions [9]. A voucher specimen was deposited in the National Museum of Marine Biology and Aquarium, Taiwan.

### 3.3. Extraction and Isolation

The freeze-dried and mince material of *Sinularia* sp. (wet weight 1.30 kg, dry weight 328 g) was extracted with ethyl acetate (EtOAc) at 25 °C (2 L × 10). The EtOAc extract left after removal of the solvent (11.4 g) was separated by silica gel and eluted using *n*-hexane/EtOAc in a stepwise fashion from 100:1 to pure EtOAc to yield 13 fractions A–M. Fraction H was separated by normal-phase HPLC (NP-HPLC) using a mixture of *n*-hexane and EtOAc (9:4) as the mobile phase to afford compounds **1** (29.5 mg) and **2** (4.4 mg).

5-Episinuleptolide acetate (**1**): [α]25D −81 (*c* 0.5, CHCl_3_); IR (neat) ν_max_ 1756, 1738, 1719 cm^−1^; ^1^H- (CDCl_3_, 400 MHz) and ^13^C- (CDCl_3_, 100 MHz) NMR data, see Table 1; ESIMS *m/z* 413 [M + Na]^+^; HRESIMS: *m/z* 413.1574 (calcd for C_2__1_H_26_O_7_ + Na, 413.1576).

Scabrolide D (**2**): [α]25D −67 (*c* 0.2, CHCl_3_); IR (neat) ν_max_ 1775, 1762, 1711 cm^−1^; ^1^H- and ^13^C-NMR spectral data were found to be in full agreement with those of reported previously [8].

### 3.4. Molecular Mechanics Calculations

Implementation of the MM2 force field [5] in CHEM3D PRO software from CambridgeSoft Corporation (Cambridge, MA, USA; ver. 9.0, 2005) was used to calculate molecular models. 

### 3.5. Cytotoxicity Testing

The cytotoxicity of norcembranoidal diterpenes **1** and **2** was assayed with a modification of the MTT [3-(4,5-dimethylthiazol-2-yl)-2,5-diphenyltetrazolium bromide] colorimetric method. Cytotoxicity assays were carried out according to previously described procedures [10,11].

## 4. Conclusions 

Cembrane- and norcembrane-type natural products are major constituents of the extracts of *Sinularia* spp. octocorals distributed in the waters off Taiwan [8,12,13,14,15,16,17,18,19,20,21,22,23,24,25,26,27,28,29,30,31,32,33,34,35]. Our continuing studies on the chemical constituents of a wild-type soft coral *Sinularia* sp. has led to the isolation of a new natural product, 5-episinuleptolide acetate (**1**), which was found to exhibit modest cytotoxicity against K562, MOLT-4, HTC-116, DLD-1, T-47D and MDA-MB-231 tumor cells. This study suggested that 5-episinuleptolide acetate (**1**) is worthy of further biomedical investigation. The study material *Sinularia* sp. has begun to be transplanted to culturing tanks with a flow-through sea water system located in the National Museum of Marine Biology and Aquarium, Taiwan for the extraction of additional natural products in order to establish a stable supply of bioactive material.

## Figures and Tables

**Table 1:** ^1^H (400 MHz, CDCl_3_) and ^13^C (100 MHz, CDCl_3_) NMR data, ^1^H–^1^H COSY and HMBC correlations for norcembranoidial diterpene **1**.

Position	*δ*_Η_ (*J* in Hz)	*δ*_C_, Mult.	^1^H–^1^H COSY	HMBC (H→C)
1	2.99 m	39.6, CH	H_2_-2, H_2_-14	C-15
2α β	2.65 m 2.52 m	45.9, CH_2_	H-1, H-2β H-1, H-2α	C-1, -3, -14, -15 C-1, -3, -14, -15
3		205.3, C		
4α β	2.53 dd (16.4, 8.8) 2.82 dd (16.4, 2.4)	43.1, CH_2_	H-4β, H-5 H-4α, H-5	C-3, -5, -6 C-3, -5, -6
5	4.27 dd (8.8, 2.4)	75.0, CH	H_2_-4	C-3, -4
6		214.5, C		
7α β	2.60 m 2.46 m	51.1, CH_2_	H-7β H-7α	C-5, -6, -8, -9, -18 C-5, -6, -8, -9, -18
8		79.0, C		
9α β	2.34 m 2.47 m	41.8, CH_2_	H-9β, H-10 H-9α, H-10	C-7, -8, -10, -11, -18 C-7, -8, -10, -11, -18
10	4.52 dt (6.8, 2.0)	80.8, CH	H_2_-9, H-11	C-8, -11, -19
11	5.47 br s	76.4, CH	H-10	C-9, -12, -13, -19, acetate carbonyl
12		127.6, C		
13	6.45 ddd (10.8, 4.4, 1.2)	147.2, CH	H_2_-14	C-1, -11, -19
14α β	2.20 ddd (14.8, 4.4, 3.6) 3.78 ddd (14.8, 10.8, 6.8)	28.4, CH_2_	H-1, H-13 H-1, H-13	C-1, -2, -12, -13, -15 C-1, -2, -12, -13, -15
15		147.0, C		
16a b	4.86 s 4.79 s	110.4, CH_2_	H-16b, H_3_-17 H-16a, H_3_-17	C-1, -15, -17 C-1, -15, -17
17	1.80 s	21.6, CH_3_	H_2_-16	C-1, -15, -16
18	1.42 s	26.5, CH_3_		C-7, -8, -9
19		167.7, C		
11-OAc	2.08 s	171.2, C20.9, CH_3_		Acetate carbonyl

**Table 2:** Cytotoxic data of norcembranoidal diterpenes **1** and **2**.

Compounds	Cell lines IC_50_ (μg/mL)					
	K562	MOLT-4	HTC-116	DLD-1	T-47D	MDA-MB-231
**1**	0.67	0.59	4.09	0.92	3.09	2.95
**2**	NA	NA	NA	NA	NA	NA
Doxorubicin *^a^*	0.15	0.01	1.11	0.22	0.40	1.30

*^a^* Doxorubicin was used as positive control. NA = not active at 20 μg/mL for 72 h.
